# The British Medical Benevolent Fund

**Published:** 1901-08-31

**Authors:** Horace Haynes


### THE BRITISH MEDICAL BENEVOLENT FUND.

Horace Haynes, Esq., M.R.C.S., writes:—

"On or about St. Luke's Day the members of the Guild of St. Luke hold their annual meeting, and have an anniversary service at St. Paul's Cathedral. I beg to suggest that a similar service be held in every town in the kingdom on that day, or on the following Sunday, and that a collection be taken at each, of which one-fifth be given to the British Medical Benevolent Fund, one-fifth to the R.M.B. College, Epsom, one-fifth to the Society for the Relief of Widows and Orphans of Medical Men, one-fifth to the *Lancet* Relief Fund, and one-fifth to church expenses; those most deserving charities would benefit greatly by these collections. St. Luke's Day, October 18th, will this year fall on a Friday."

